# GPD1L inhibits renal cell carcinoma progression by regulating PINK1/Parkin‐mediated mitophagy

**DOI:** 10.1111/jcmm.17813

**Published:** 2023-06-29

**Authors:** Ting Liu, Hengcheng Zhu, Minghuan Ge, Zhou Pan, Yan Zeng, Yan Leng, Kang Yang, Fan Cheng

**Affiliations:** ^1^ Department of Urology Renmin Hospital of Wuhan University Wuhan China

**Keywords:** mitophagy, phosphate dehydrogenase 1‐like, PINK1/Parkin pathway, renal cell carcinoma

## Abstract

Few approaches have been conducted in the treatment of renal cell carcinoma (RCC) after nephrectomy, resulting in a high mortality rate in urological tumours. Mitophagy is a mechanism of mitochondrial quality control that enables selective degradation of damaged and unnecessary mitochondria. Previous studies have found that glycerol‐3‐phosphate dehydrogenase 1‐like (GPD1L) is associated with the progression of tumours such as lung cancer, colorectal cancer and oropharyngeal cancer, but the potential mechanism in RCC is still unclear. In this study, microarrays from tumour databases were analysed. The expression of GPD1L was confirmed by RT–qPCR and western blotting. The effect and mechanism of GPD1L were explored using cell counting kit 8, wound healing, invasion, flow cytometry and mitophagy‐related experiments. The role of GPD1L was further confirmed in vivo. The results showed that GPD1L expression was downregulated and positively correlated with prognosis in RCC. Functional experiments revealed that GPD1L prevented proliferation, migration and invasion while promoting apoptosis and mitochondrial injury in vitro. The mechanistic results indicated that GPD1L interacted with PINK1, promoting PINK1/Parkin‐mediated mitophagy. However, inhibition of PINK1 reversed GPD1L‐mediated mitochondrial injury and mitophagy*.* Moreover, GPD1L prevented tumour growth and promoted mitophagy by activating the PINK1/Parkin pathway in vivo. Our study shows that GPD1L has a positive correlation with the prognosis of RCC. The potential mechanism involves interacting with PINK1 and regulating the PINK1/Parkin pathway. In conclusion, these results reveal that GPD1L can act as a biomarker and target for RCC diagnosis and therapy.

## 1 INTRODUCTION

Renal cell carcinoma (RCC) is among the top three genitourinary malignancies, with more than 400,000 diagnosed cases and 180,000 deaths each year.^1^ Most renal cell cancers are clear cell renal cell carcinomas (ccRCCs), which account for 60%–65% of all renal cell cancers. Papillary RCCs (pRCCs), meanwhile, account for 13%–15% of RCCs.^2^ Despite the rapid development of imaging technology, close to 30% of RCC individuals have local or distant metastasis at the time of diagnosis.^3^ Currently, the postsurgical treatment efficacy for patients with RCC remains unacceptable due to its insensitivity to conventional treatment.

Autophagy is a naturally conserved self‐degradative process that removes needless or dysfunctional metabolic components through autophagosome‐dependent regulatory mechanisms.^4^ Mitophagy is a mechanism of mitochondrial quality control that enables selective degradation of damaged and unnecessary mitochondria and avoids disadvantageous stimuli and conditions such as starvation, oxidative stress and mitochondrial transmembrane potential loss.^5^, ^6^ Abnormal and dysfunctional mitophagy events are linked with a series of pathological processes, including tumorigenesis and cardiovascular and chronic kidney disease.^7^, ^8^

Glycerol‐3‐phosphate dehydrogenase 1‐like (GPD1L) was first reported in the mammalian gene sequence in 2002.^9^ The GPD1L protein is expressed in the cytoplasm and linked with the plasma membrane, where it binds to the sodium channel. The mutation of GPD1L has been correlated with Brugada syndrome and sudden infant death syndrome.^10^, ^11^ Studies have shown that GPD1L also plays an important role in the regulation of cardiac sodium current, colorectal cancer and oropharyngeal cancer.^12^ However, the function and mechanism of GPD1L in RCC remains unknown.

In the present study, the relationship between GPD1L and RCC patients was explored through bioinformatics analysis of data derived from TCGA database. Functional and mechanistic studies revealed that GPD1L suppressed mitophagy through the PINK1/Parkin signalling pathway in RCC progression. Our study demonstrated that GPD1L might be a promising potential target for the treatment of RCC patients.

## 2 MATERIALS AND METHODS

### 2.1 Data preprocessing and patient inclusion

RNA‐seq data of RCC derived from TCGA database and the GEO database (GSE16441, GSE36895, GSE53757, GSE66270, GSE68417) were analysed using the Limma package.^13^ Differentially expressed genes (DEGs) with |log2‐fold change| > 2 and *p* value <0.05 were included.

### 2.2 Cell lines and transfection

Human RCC cell lines such as 786‐O, 769‐P, ACHN and Caki‐1 and the normal human renal cell line HK2 were purchased from the China Centre for Type Culture Collection (Wuhan, China). All cells were cultured according to the manufacturers' instructions. Lentivirus overexpressing GPD1L and the corresponding vector purchased from GeneCreate Company (Wuhan, China) were transfected into ACHN and 786‐O cells using Lipofectamine 2000 reagent (Thermo Fisher Scientific, USA) according to the manufacturers' instructions. GPU6/Hygro‐based PINK1 knockdown vectors were purchased from GeneCreate Company. The RNA sequences were as follows: si‐PINK1: 5′‐GTATGTGCCTTGAACTGAATA‐3′; si‐NC, 5′‐AATTCTCCGAACGTGTCACGT‐3′. The transfection efficiency of cells was further detected through RT–qPCR and western blotting.

### 2.3 Cell proliferation assay

To evaluate cell proliferation, 769‐P and ACHN cells were seeded into 96‐well plates and incubated with CCK8 according to the manufacturer's instructions. Then, the optical density of each plate was measured at 450 nm.

### 2.4 Cell proliferation, migration and invasion

A bromodeoxyuridine (BrdU) assay (Abcam, USA) was used to test cell proliferation ability following the manufacturer's protocols. Briefly, 769‐P and ACHN cells were seeded into 6‐well plates under BrdU‐labelling solution for 24 h at 37°C in a CO_2_ incubator. Then, the cells were fixed and permeabilized according to the standard immunocytochemistry and photographed under a microscope. A wound healing assay was used to test the capacity of cell migration. Briefly, 769‐P and ACHN cells were seeded into six‐well plates and incubated with serum‐free medium. A 200‐μl plastic pipette tip was utilized to make a scratch after the density of the cells reached 80%, and the migration distance was photographed after 24 h. A Matrigel chamber (BD Biosciences, USA) was used to detect cell invasion ability according to the manufacturer's protocols. Briefly, cells were seeded into the upper chamber in serum‐free medium, and the lower chamber was filled with 10% fetal bovine serum. After 24 h, the non‐migrated cells on the upper side of the chamber were removed and stained with crystal violet. Finally, the cells were photographed and analysed under a microscope.

### 2.5 Western blot

Western blot assays were performed as previously described.^14^ Briefly, proteins were extracted from cells and tissue in protein lysis buffer (Servicebio, China) and tested using a BCA Protein Assay kit (Thermo Fisher Scientific, USA). Then, proteins were denatured and separated using SDS–PAGE, and transferred into polyvinylidene fluoride (PVDF) membranes under transfer buffer. After blocking with 0.5% nonfat milk, the PVDF membranes were incubated with primary antibodies (Table S1) overnight at 4°C. After washing three times in Tris‐buffered saline‐Tween buffer, the membranes were incubated with horseradish peroxidase‐linked anti‐rabbit/mouse secondary antibody at a 1:5000 dilution (Cell Signaling Technology, USA) and visualized using an ECL protein‐detection system (Odyssey, USA).

### 2.6 Flow cytometry analysis of apoptosis

The transfected 769‐P and ACHN cells were seeded into 6‐well plates under 10% fetal bovine serum. After 24 h, the cells were collected and stained with Annexin V‐FITC and propidium iodide (PI) using a flow cytometry detection system according to the manufacturer's protocols (BD Biosciences, USA).

### 2.7 Detection of reactive oxygen species

Mitochondrial reactive oxygen species (MitoSOX) were tested using MitoSOX Red Mitochondrial Superoxide Indicators (Thermo Fisher, USA) according to the manufacturer's protocols. Briefly, RCC cells were incubated with MitoSOX Red dilutions for 10 min and analysed with a cytometry detection system.

### 2.8 Transmission electron microscopy

Cells were seeded into 6‐well plates, fixed in 4% formaldehyde and 1% glutaraldehyde buffer (pH 7.2), and postfixed in 1% phosphate‐buffered osmium. Then, all cells were dehydrated, embedded in a beam capsule, sectioned and stained. Finally, each sample was photographed using transmission electron microscopy (Hitachi, Japan).

### 2.9 Immunofluorescence and immunohistochemistry

Cells were seeded into six‐well plates and fixed with 4% paraformaldehyde (pH 7.4) for 15 min. After permeabilization, cells were incubated with primary antibodies (Table S1) overnight at 4°C, followed by secondary antibody (Cell Signaling Technology, USA). The nuclei of RCC cells were stained with DAPI. JC‐1 staining was performed based on the manufacturers' instructions. For immunohistochemistry, 4 μm sections of paraffin‐embedded mouse tumours were deparaffinized, and incubated with primary antibodies (Table S1) at 4°C overnight. After washing, each sample was incubated with HRP‐conjugated secondary antibody for 30 min and stained with 3,3‐diaminobenzidine tetrahydrochloride dilution (Maixin, China). Each sample was then photographed using a microscope (Olympus, Japan).

### 2.10 Real‐time quantitative PCR

Total RNA was extracted from samples using TRIzol reagent (Sigma Aldrich, USA) and detected using a spectrophotometer (NanoDrop, USA). Then, the RNAs were transcribed into complementary cDNA. Finally, RT–qPCR examination was performed using SYBR Green mix (Thermo Fisher Scientific, USA). The primer sequences are shown in Table S2.

### 2.11 Tumour xenograft model

All experimental procedures involving animals were approved by the ethics committee of Animal Care and Treatment of Renmin Hospital of Wuhan University. Male BALB/c nude mice (5 weeks, 20–25 g) were purchased from the Charles River Animal Technology Company (Beijing, China) and randomly divided into two groups (*n* = 5 per group). Then, 786‐O cells (1.0 × 10^7^ cells/mouse) overexpressing GPD1L or vector were directly injected into the subcutaneous tissue of the mouse thigh. After 28 days, the mice were sacrificed, and the tumours were removed for further analysis.

### 2.12 Statistical analysis

SPSS 24.0 software (IBM, USA) was utilized for statistical analysis. Three replicates were performed for experiments in this study, and the data are shown as the mean ± standard deviation (SD). Differences between two groups and multiple groups were analysed using Student's *t*‐test and one‐way anova, respectively. The Kaplan–Meier method was performed for patient survival in RCC. *p* < 0.05 was considered significant.

## 3 RESULTS

### 3.1 GPD1L expression is downregulated and positively correlated with prognosis in RCC

**FIGURE 1:**
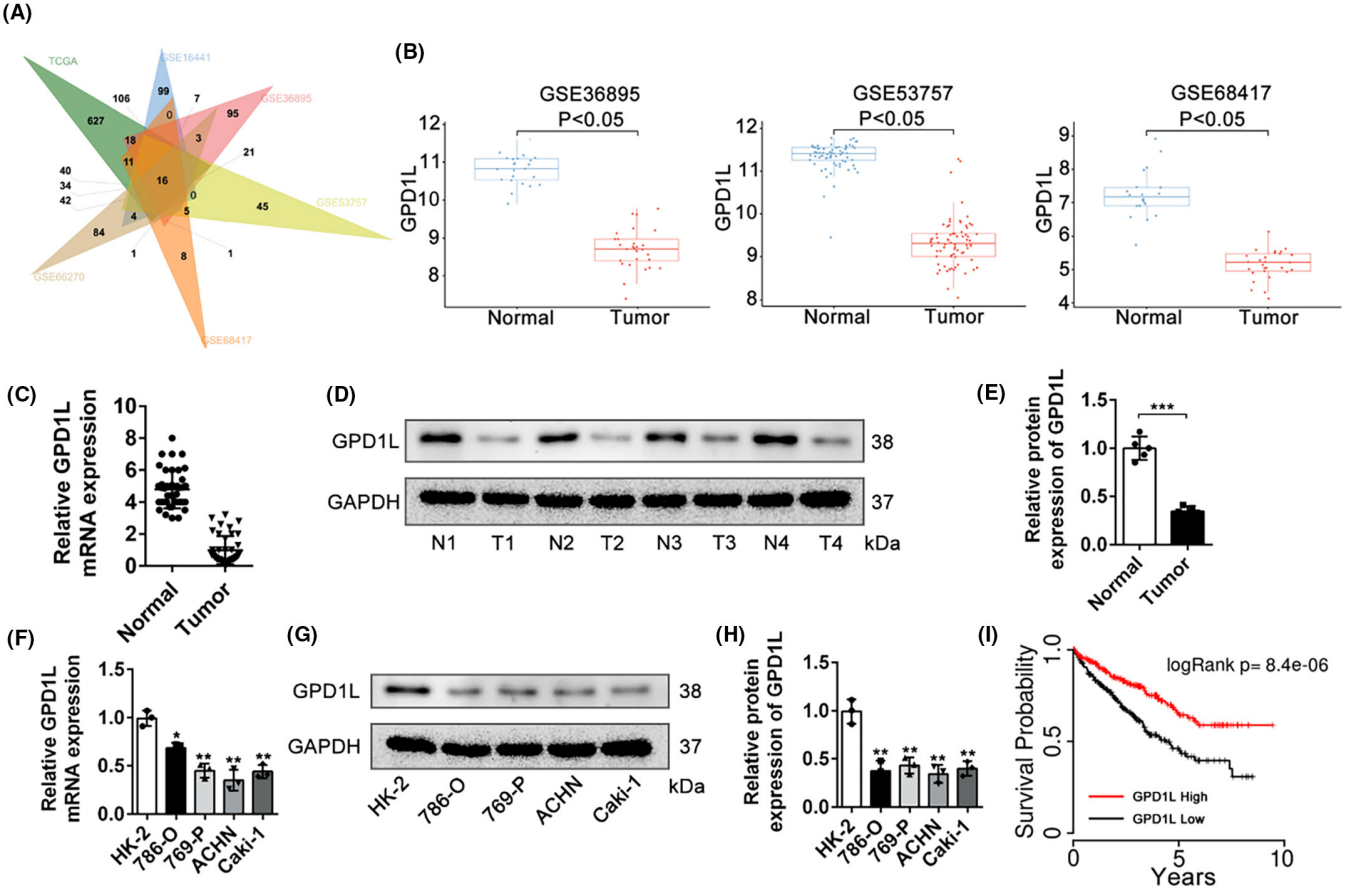
Glycerol‐3‐phosphate dehydrogenase 1‐like (GPD1L) expression is downregulated and positively correlated with prognosis in renal cell carcinoma. (A) Interaction analysis of TCGA and GEO datasets. (B) GDP1L expression in the GSE36895, GSE53757 and GSE68417 datasets. (C) Comparison of GDP1L expression between adjacent nontumour and tumour tissue from clinical samples as tested by RT–qPCR. (D,E) Western blots and quantitative analysis of GPD1L between adjacent nontumour and tumour tissue, *n* = 5. (F) RT–qPCR analysis of GPD1L expression in normal and tumour cells, *n* = 3. (G,H) Western blots and quantitative analysis of GPD1L expression in normal and tumour cells. (I) Kaplan–Meier survival curve of GPD1L based on the TCGA dataset. *p < 0.05, **p < 0.01, ***p < 0.001 versus normal or HK2 cells.

After calculating differentially expressed genes (Figure S1) and interacting genes (Figure 1A) and performing a survival analysis (Figure S2, Figure 1I) to rank the significant genes, the results of our bioinformatics analysis showed that GPD1L played an important role in RCC. As shown in Figure 1B, compared with the tumour group, the expression of GPD1L in the normal group was much higher in the GEO datasets. To further verify the bioinformatic results, 40 pairs of RCC and adjacent nontumour kidney tissues were collected and validated by RT–qPCR and western blotting. The results consistently indicated that the expression of GPD1L was decreased in kidney tumour samples and tumour cell lines compared with adjacent nontumour kidney tissues and normal kidney cells at both the mRNA and protein levels (Figure 1C–H). These results showed that the expression of GPD1L was decreased in RCC, and GPD1L was associated with advanced disease in RCC, revealing that GPD1L may play an important role in RCC.

### 3.2 GPD1L prevents proliferation, migration and invasion, and promotes apoptosis of RCC cells

**FIGURE 2:**
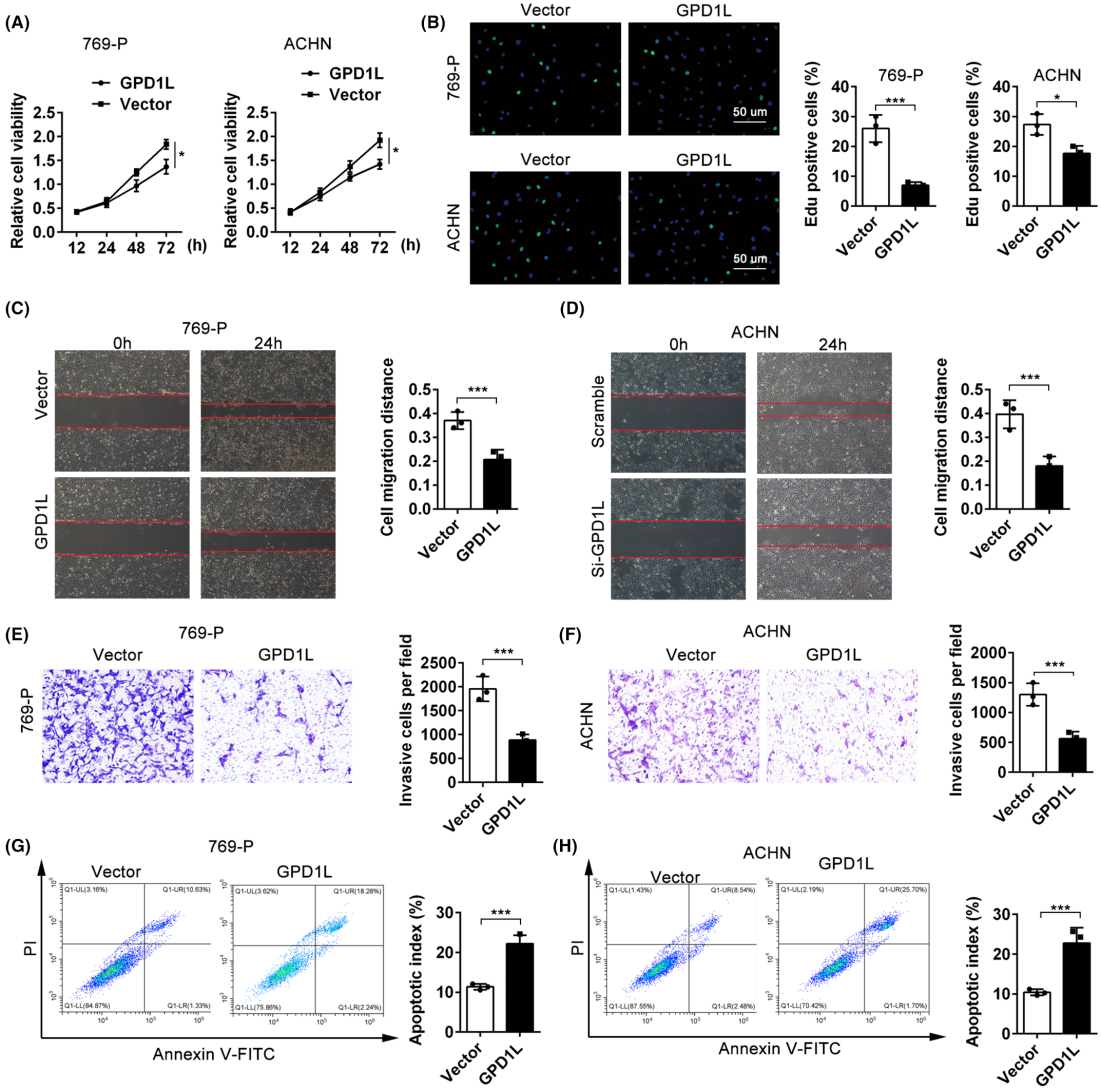
Glycerol‐3‐phosphate dehydrogenase 1‐like (GPD1L) is important for renal cell carcinoma tumour progression. (A) 769‐P and ACHN cells transfected with vector or GPD1L‐overexpressing lentivirus for 12, 24, 48 and 72 h, and the proliferative viability was detected by CCK8 assay. (B) Bromodeoxyuridine analysis of 769‐P and ACHN cells transfected with vector or GPD1L overexpression vector. (C,D) Wound‐healing migration assays and quantitative analysis of the open areas between vector‐ and GPD1L‐overexpressing cells. Scale bars, 200 μm. (E,F) The migration ability of renal cell carcinoma cells was tested by transwell assay. (G,H) Flow cytometry analysis of apoptosis in 769‐P and ACHN cells transfected with vector or GPD1L‐overexpressing lentivirus. *p < 0.05, **p < 0.01, ***p < 0.001 versus the vector group, each group *n* = 3.

To explore the potential biological function of GPD1L, the transfection efficiency of GPD1L overexpression was confirmed using RT–qPCR and western blotting in 769‐P and ACHN cells (Figure S3), followed by functional experiments. The results of the CCK8 assay showed that overexpression of GPD1L in both 769‐P and ACHN cells significantly suppressed the capacity for cell proliferation, with decreased cell viability (Figure 2A). Similar results were exhibited in the BrdU assay, which revealed that the number of BrdU‐positive cells in the GPD1L‐overexpressing group was decreased compared to that in the vector group (Figure 2B). The wound healing assay indicated that in both 769‐P and ACHN cells, higher GPD1L levels decreased the migration ability of cells compared to the vector groups (Figure 2C,D). The transwell assay displayed consistent results of wound healing (Figure 2E,F). Moreover, a flow cytometry experiments revealed that GPD1L overexpression resulted in significant apoptosis of cells compared to the control groups (*p* < 0.001, Figure 2G,H). Together, these results illustrate that GPD1L suppresses proliferation, invasion and apoptosis in the progression of RCC.

### 3.3 GPD1L induces mitochondrial depolarization

**FIGURE 3:**
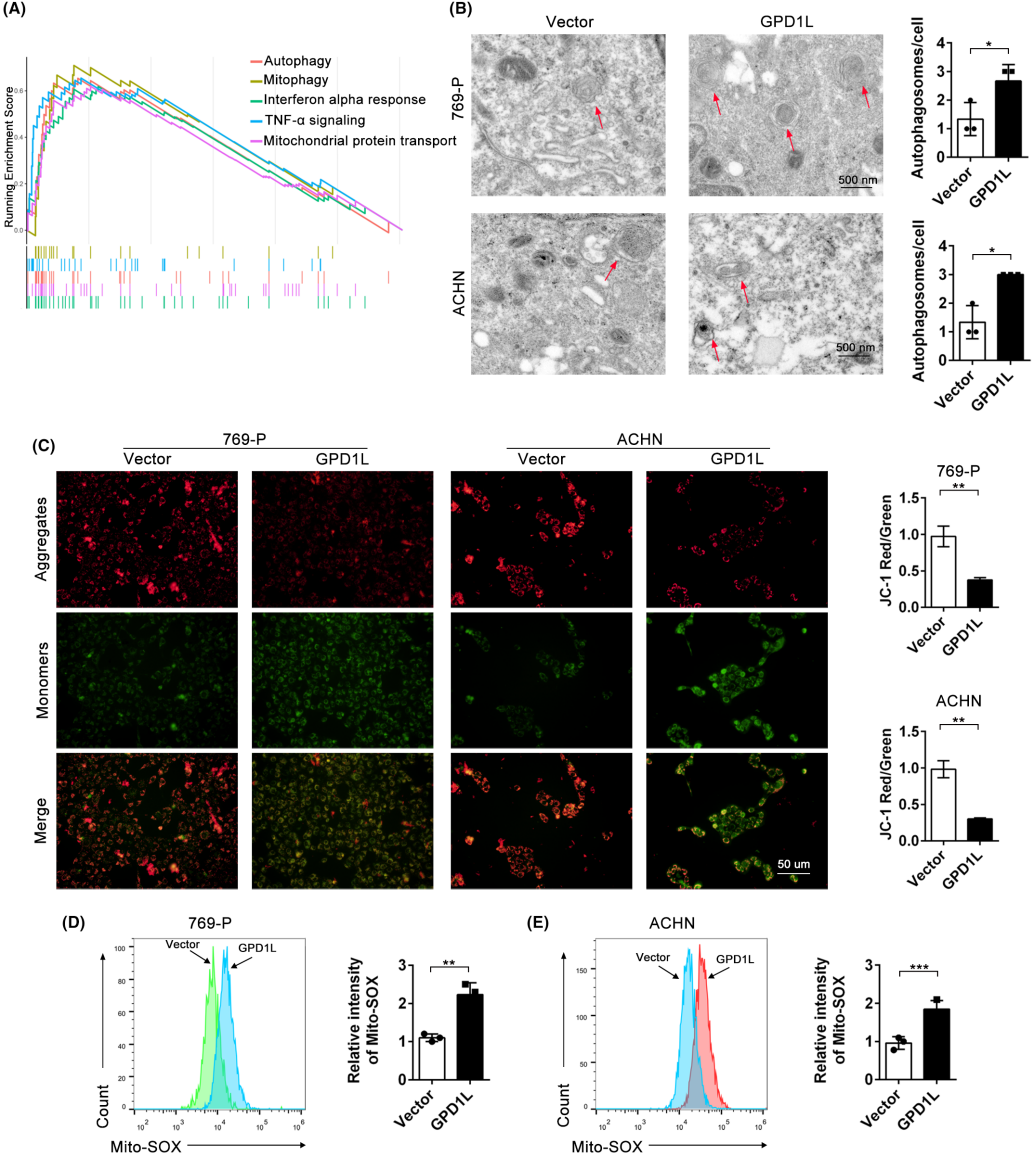
Glycerol‐3‐phosphate dehydrogenase 1‐like (GPD1L) increases mitochondrial membrane potential. (A) GSEA of significant pathways based on GPD1L expression in The Cancer GenomicAtlas renal cell carcinoma (RCC) datasets. (B) Electron micrographs of autophagosomes (red arrows) in RCC cells transfected with vector or GPD1L‐overexpressing lentivirus. (C) JC‐1 staining and quantitative analysis of the intracellular mitochondrial membrane potential of 769‐P and ACHN cells. (D,E) Intracellular mitochondrial superoxide levels of 769‐P and ACHN cells were tested using MitoSOX through flow cytometry. *p < 0.05, **p < 0.01, ***p < 0.001 versus the vector group, each group *n* = 3.

To further investigate the potential mechanisms of GPD1L in RCC, GSEA basing on the expression of GPD1L in TCGA database was utilized based on RNA‐seq data. As indicated in Figure 3A, the top five important signalling pathways were autophagy, mitophagy, interferon alpha response, TNF‐α signalling and mitochondrial protein transport. Then, transmission electron microscopy was performed to test the ultrastructure of RCC cells. As shown in Figure 3B, GPD1L induced the accumulation of many autophagosomes in both 769‐P and ACHN cells, displaying double‐membrane vesicles (marked with red arrows). Moreover, compared with the control cells, GPD1L overexpression resulted in the depolarization of mitochondrial membrane potential (Figure 3C). MitoSOX is a crucial indicator of mitochondrial activity because it reflects the progression of oxidative phosphorylation.^15^ As shown in Figure 3D, overexpressing GPD1L increased the levels of mitochondrial reactive oxygen species compared with the controls. These results show that overexpressing GPD1L facilitates mitochondrial membrane potential depolarization and induces mitochondrial injury.

### 3.4 GPD1L promotes mitophagy by regulating the PINK1/Parkin pathway in vitro

**FIGURE 4:**
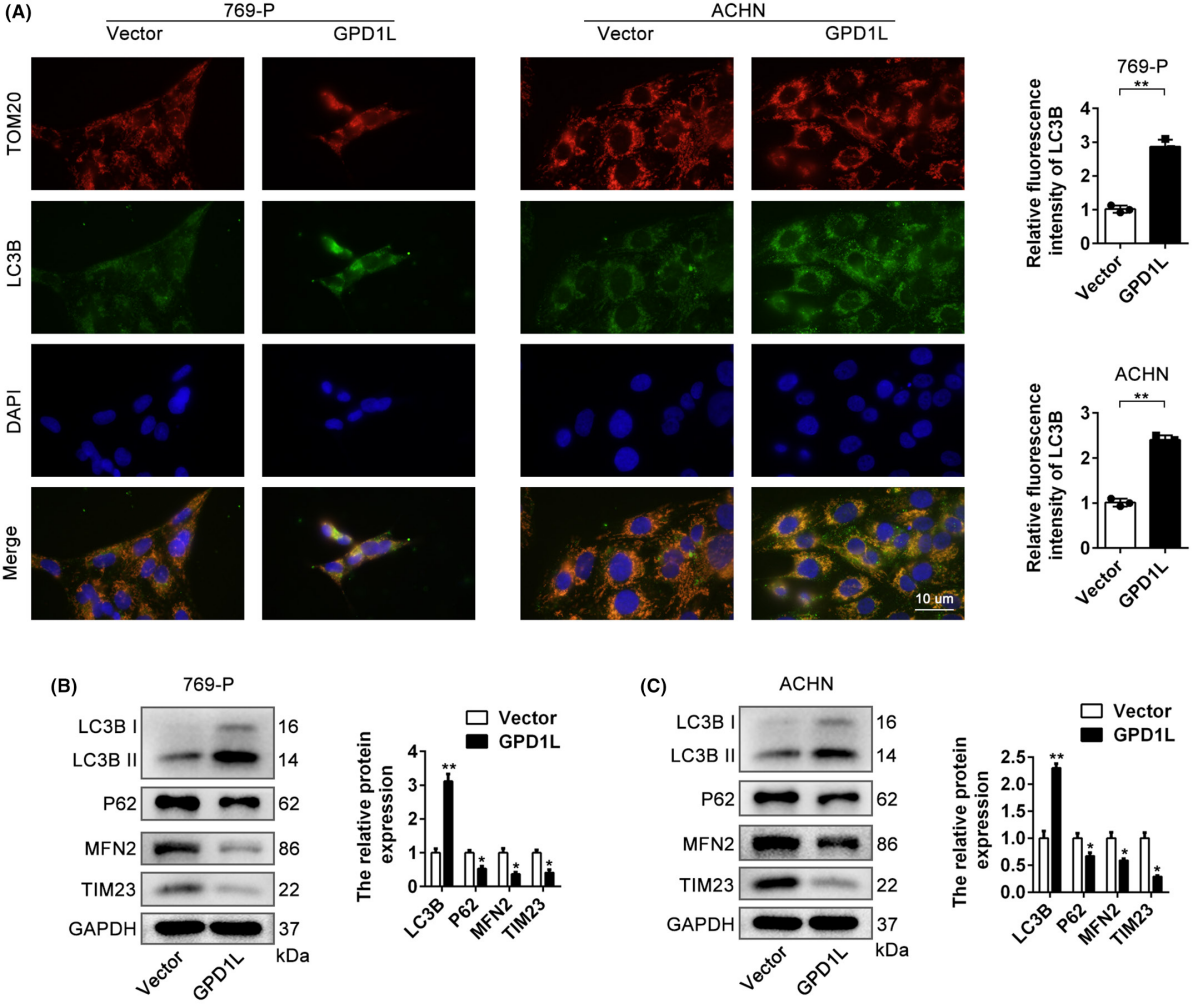
Glycerol‐3‐phosphate dehydrogenase 1‐like (GPD1L) facilitates mitophagy in vitro. (A) Representative immunofluorescence images of TOM20 (red) and LC3BII (green) expression in 769‐P and ACHN cells infected with either vector or GPD1L‐overexpressing lentivirus. (B,C) Representative western blots and quantitative analysis of mitophagy‐related proteins in 769‐P and ACHN cells infected with either vector or GPD1L‐overexpressing lentivirus. *p < 0.05, **p < 0.01 versus the vector group, each group *n* = 3.

To determine the role of GPD1L in mitophagy, we measured the expression of LC3B and TOM20 in vitro. As shown in Figure 4A, immunofluorescence revealed that LC3B was mainly colocalized with the mitochondrial marker TOM20 in 769‐P and ACHN cells (yellow), and the presence of GPD1L led to increased expression of LC3B (green). Moreover, the results of western blotting indicated that compared to the vector cells, the expression of LC3B II was significantly elevated, but the expression levels of P62, MFN2 and TIM23 were decreased in the GPD1L‐overexpressing group in both 769‐P and ACHN cells (Figure 4B,C). These results suggest that overexpressing GPD1L facilitates mitophagy in the progression of RCC.

### 3.5 GPD1L colocalizes with PINK1

**FIGURE 5:**
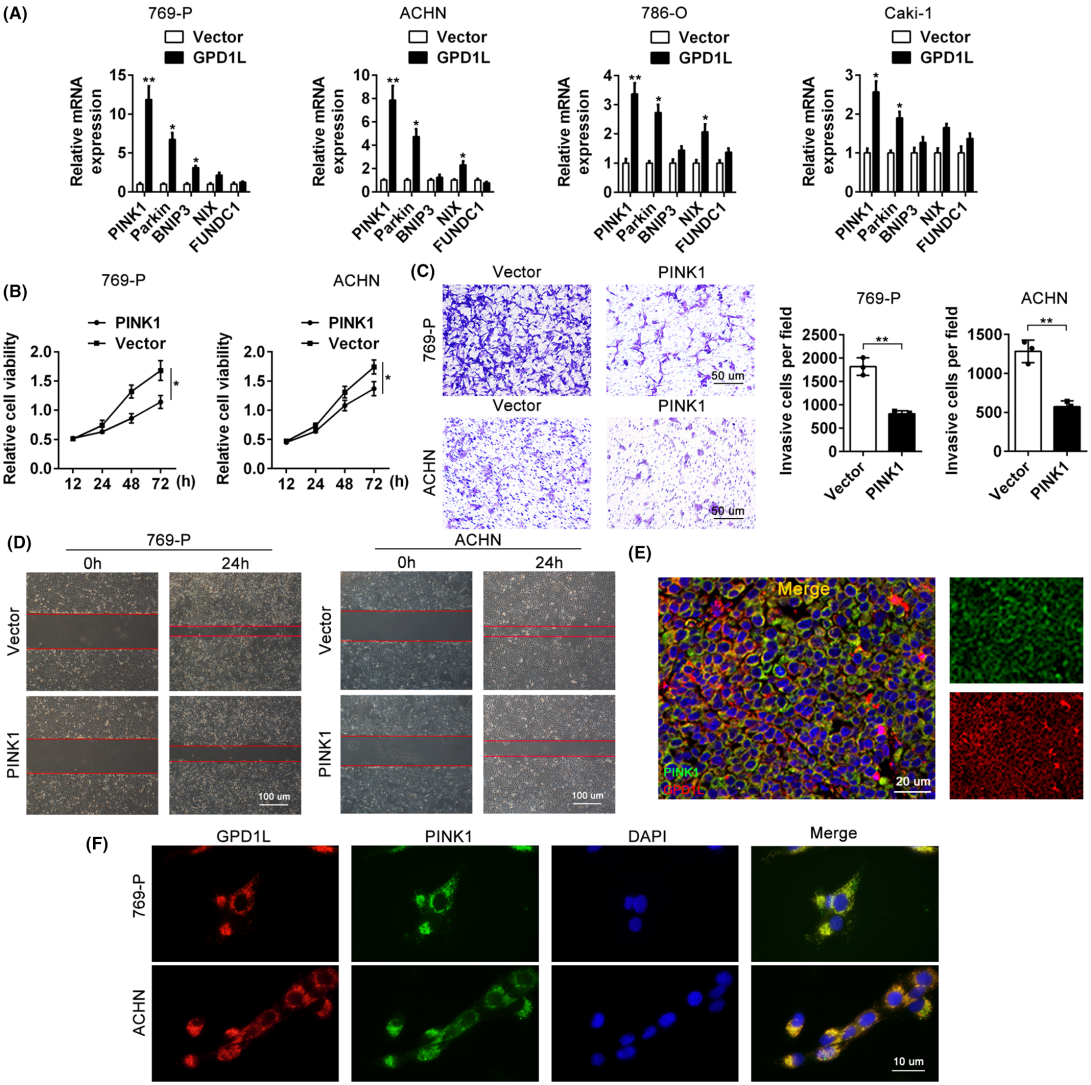
Glycerol‐3‐phosphate dehydrogenase 1‐like (GPD1L) interacts with PINK1. (A) Representative RT–qPCR analysis of PINK1, Parkin, BNIP3, NIX and FUNDC1 levels in renal cell carcinoma (RCC) cells infected with either vector or GPD1L‐overexpressing lentivirus. (B) Representative CCK8 analysis of cellular proliferative ability in 769‐P and ACHN cells transfected with either vector or PINK1‐overexpressing lentivirus. (C) The migration ability of RCC cells transfected with either vector or PINK1 overexpression was tested by transwell assay and quantitative analysis. Scale bars, 50 μm. (D) Wound healing migration assays and quantitative analysis of the open areas in 769‐P and ACHN cells. Scale bars, 100 μm. (E) Representative co‐immunoprecipitation analysis between PINK1 and GPD1L in RCC cells. (F) The distribution and colocalization of GPD1L and PINK1 were analysed in vitro and in vivo. Scale bars, 10 μm. *p < 0.05, **p < 0.01 versus the vector group, each group *n* = 3.

The PINK1/Parkin‐mediated signalling pathway is usually documented as the focus of mitophagy.^16^ To investigate the mechanism of GDP1L, the main genes PINK1, Parkin, BNIP3, NIX and FUNDC1 involved in the PINK1/Parkin pathway were measured using RT–qPCR. As indicated in Figure 5A, the levels of PINK1, Parkin, were significant increase in the GDP1L‐transfection group, while the alterations in BNIP3, NIX mRNA were not obvious compared to the vector group in 769‐P, ACHN, 768‐O and Caki‐1 cells. To our surprise, the proteins of BNIP3, NIX and FUNDC1 were no significant change, but a remarkable increased in PINK1 and Parkin protein levels (Figure S4A,B). Then, we tested the transfection efficiency of PINK1 overexpression by RT–qPCR and western blotting in 769‐P and ACHN cells (Figure S4C–E), followed by functional experiments in vitro. The CCK8 assay revealed that PINK1 repressed proliferation, with lower cell viability (Figure 5B), compared to the vector cells. Consistent with the CCK8 findings, the transwell and wound healing assays showed that PINK1 overexpression resulted in suppressive capacities of cellular migration and invasion, with fewer cells and more invasive distances compared to the controls (Figure 5C,D). Moreover, we detected the distribution of GPD1L (red) and PINK1 (green) in both 769‐P and ACHN cells by confocal microscopy, which showed that GPD1L colocalized with PINK1 (yellow, Figure 5F). A similar colocalization phenomenon was also observed in vivo (Figure 5E). These data indicate that GPD1L may interact with PINK1, promoting mitophagy in RCC.

### 3.6 Suppression of PINK1 restores GPD1L‐mediated mitochondrial membrane potential and mitophagy in vitro

**FIGURE 6:**
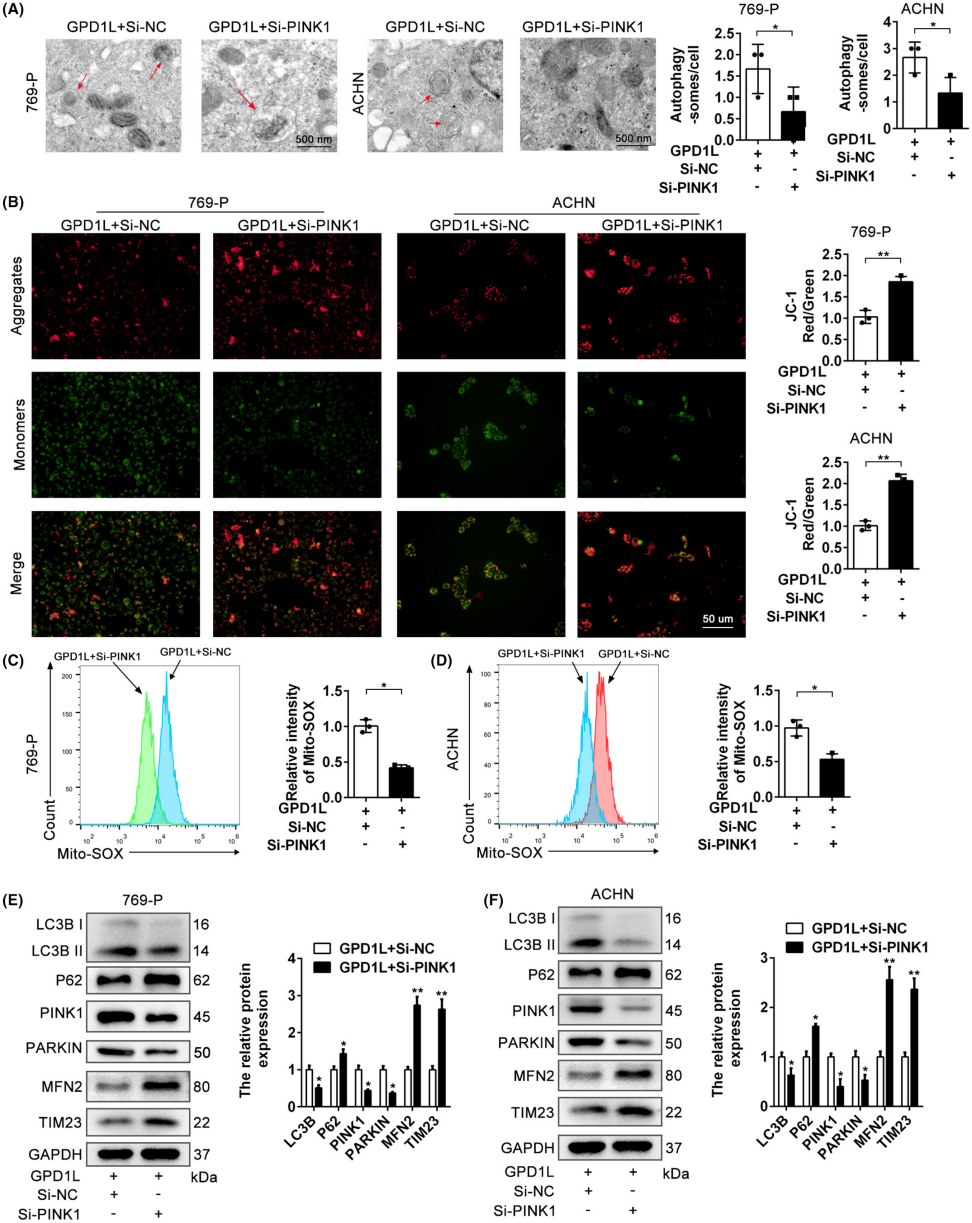
Inhibition of PINK1 restored Glycerol‐3‐phosphate dehydrogenase 1‐like (GPD1L)‐mediated mitochondrial membrane potential and mitophagy. (A) Electron micrographs of autophagosomes (red arrows) and quantitative analysis in 769‐P and ACHN cells overexpressing GPD1L transfected with either si‐NC or si‐PINK1. (B) JC‐1 staining and quantitative analysis of the intracellular mitochondrial membrane potential of GPD1L 769‐P and ACHN cells stably overexpressing GPD1L transfected with either si‐NC or si‐PINK1. (C,D) Intracellular mitochondrial superoxide levels of 769‐P and ACHN cells were tested using MitoSOX through flow cytometry for the indicated groups. (E,F) Representative western blots and quantitative analysis of mitophagy‐related proteins in 769‐P and ACHN cells. *p < 0.05, **p < 0.01 versus the vector group, each group *n* = 3.

To further uncover the potential correlation between GPD1L and PINK1, 769‐P and ACHN cells overexpressing GPD1L were transfected with si‐NC (Scramble) or si‐PINK1, and the transfection efficiency was confirmed (Figure S5). As shown in Figure 6A, PINK1 knockdown reversed the number of autophagosomes (red arrow) mediated by GPD1L. The JC‐1 staining assay indicated that Si‐PINK1 resulted in increased JC‐l red staining and decreased green staining (Figure 6B), indicating that Si‐PINK1 restores the depolarization of mitochondrial membrane potential caused by GPD1L. Consistent with JC‐1 findings, the levels of MitoSOX were also decreased in the si‐PINK1 group (Figure 6C,D), mitigating the mitochondrial damage injury by GPD1L overexpression. The results of western blotting showed that compared to the scramble group, the expression of LC3BII was significantly decreased, but the expression levels of p62, PINK1, Parkin, MFN2 and TIM23 were increased in the si‐PINK1 group (Figure 6E,F). These results illustrate that PINK1 can reverse the effects of mitochondria and mitophagy triggered by GPD1L.

### 3.7 GPD1L prevents tumour growth and promotes mitophagy in vivo

**FIGURE 7:**
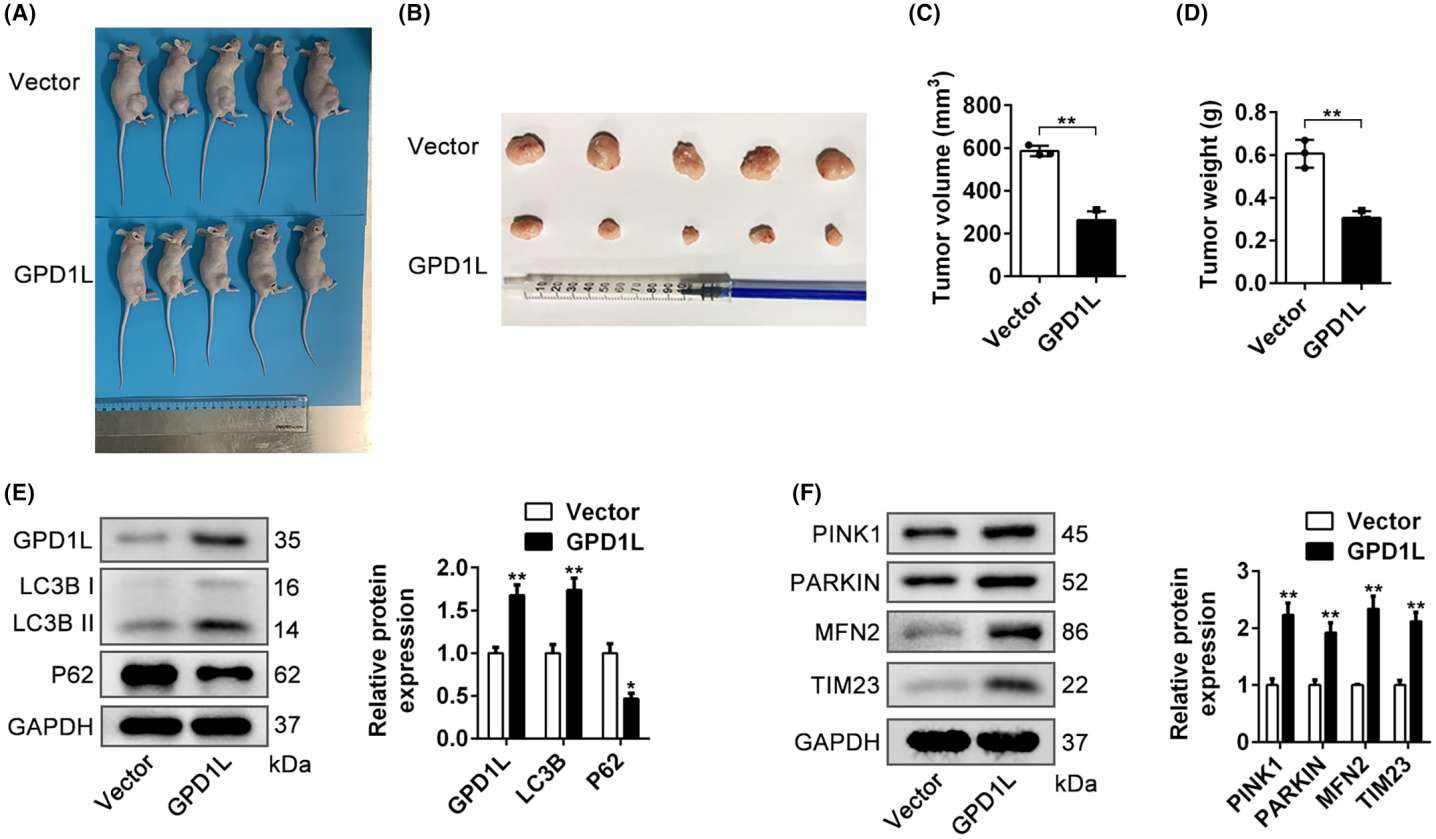
Glycerol‐3‐phosphate dehydrogenase 1‐like (GPD1L) prevents tumour growth and promotes mitophagy in vivo. (A) Representative images of the subcutaneous xenograft model between the vector group and the GPD1L‐overexpressing group. (B–D) Representative images of gross appearance, tumour volume and weight of tumours for the displayed groups, *n* = 5. (E,F) Representative western blots and quantitative analysis of mitophagy‐related proteins in the indicated groups. *p < 0.05, **p < 0.01 versus the vector group, *n* = 3.

To investigate the role of GPD1L in the tumour burden, a subcutaneous xenograft model was established with vector or GPD1L stably overexpressed in 769‐P cells (Figure 7A). As indicated in Figure 7B–D, compared with the vector group, the tumour volume and tumour weight were significantly decreased in the GPD1L‐overexpressing group. The results of western blot analyses revealed that overexpressing GPD1L increased the expression levels of LC3B, PINK1, Parkin, but decreased the expression of p62, MFN2 and TIM23 (Figure 7E,F). These results illustrate that GPD1L inhibits tumour growth by promoting mitophagy.

## 4 DISCUSSION

Mitophagy, a highly regulated multistep procedure of selective removal of dysfunctional or damaged mitochondria, is crucial for maintaining the metabolic balance of mitochondrial homeostasis in the process of cell development,^17^ However, excessive mitophagy can lead to mitochondrial dysfunction, bioenergetic deficits and subsequent cell death.^18^ A growing number of studies have documented that the PINK1/Parkin pathway plays a crucial role in the progression of tumorigenesis.^19^, ^20^ The results from clinical cohorts showed that PINK1 was significantly positively correlated with patients and may serve as an independent predictor for overall survival in kidney cancer.^21^ In addition, PINK1 could suppress the growth and invasion of cells, while loss of PINK1 resulted in the Warburg effect by promoting the elevation of hypoxia‐inducible factor‐1 and reactive oxygen species in glioblastoma.^22^ Mutation of Parkin was identified in colorectal cancer samples, and overexpression of Parkin blocked the proliferative ability of colorectal tumour cells.^23^ Consistent with the discoveries in a previous study, the results of the present study showed that overexpressing PINK1 inhibited the proliferation, invasion and migration capacities in both 769‐P and ACHN cells.

Over the past decades, few approaches have been conducted in the treatment of RCC after nephrectomy, resulting in a high mortality rate in urological tumours.^24^ With in‐depth research on the mechanism of kidney cancer development, many applicable targeted treatments for patients have been developed. A few studies have identified the role of GPD1L in the progression of tumours, such as lung cancer, colorectal cancer and oropharyngeal cancer, but the potential mechanism in RCC is still unclear. The results of our study from kidney tumour samples and adjacent normal samples showed that the expression of GPD1L was decreased in RCC and positively correlated with prognosis in RCC. To further investigate the underlying mechanisms of GPD1L in RCC, we tested mitophagy‐related genes using RT–qPCR. To our surprise, the levels of PINK1, Parkin, BNIP3 and NIX were significantly increased in the GDP1L‐transfection group, and the alteration of PINK1 expression was highest. PINK1 is a mitochondrial serine/threonine kinase that acts as an effective sensor of mitochondrial quality control.^25^ Mitochondrial malfunction lacks sufficient membrane potential to import PINK1 into the inner mitochondrial membrane, leading to Parkin recruitment to target injured mitochondria for cleavage by mediating mitophagy.^26^ Consistent with previous studies, our results also indicated that PINK1 played a crucial role in regulating RCC. Moreover, GPD1L may interact with PINK1, promoting mitophagy, and inhibition of PINK1 could reverse GPD1L‐mediated mitochondrial membrane potential depolarization and mitophagy. Similar findings in vivo also supported this concept, showing that GPD1L suppressed tumour growth by promoting PINK1‐mediated mitophagy.

In conclusion, our study shows that GPD1L can act as an inhibitor of RCC tumorigenesis and is positively correlated with the prognosis of RCC patients. The potential mechanism may involve interacting with PINK1 and regulating the PINK1/Parkin pathway, which prevents RCC progression. However, it is unclear whether GPD1L can directly affect PINK1, and the exact mechanism of the interaction between GPD1L and PINK1 will be further explored in the future.

## AUTHOR CONTRIBUTIONS

**Ting Liu:** Conceptualization (equal); data curation (equal); writing – original draft (equal). **Hengcheng Zhu:** Conceptualization (equal); supervision (equal); visualization (equal); writing – review and editing (equal). **Minghuan Ge:** Conceptualization (equal); visualization (equal); writing – review and editing (equal). **Zhou Pan:** Conceptualization (equal); data curation (equal); formal analysis (equal). **Yan Zeng:** Conceptualization (equal); data curation (equal); formal analysis (equal). **Yan Leng:** Supervision (equal); validation (equal). **Kang Yang:** Conceptualization (equal); data curation (equal); investigation (equal). **Fan Cheng:** Supervision (equal); validation (equal); visualization (equal); writing – review and editing (equal).

## FUNDING INFORMATION

This work was supported by the National Natural Science Foundation of China (No. 82100703), the Natural Science Foundation of Hubei Province (No. 2022CFC015) and the Funds project for young teachers of Wuhan University in Central Universities (No. 2042021kf0099).

## CONFLICT OF INTEREST STATEMENT

The authors declare no conflicts of interest.

## Supporting information


Figure S1

Figure S2

Figure S3

Figure S4

Figure S5

Table S1

Table S2

## Data Availability

The data that support the findings of this study are available on request from the corresponding author. The data are not publicly available due to privacy or ethical restrictions.
