# Supplementary figures and images for: GPD1L inhibits renal cell carcinoma progression by regulating PINK1/Parkin‐mediated mitophagy
Source: J Cell Mol Med. 2023 Jun 29;27(16):2328–39. doi: 10.1111/jcmm.17813 (PMC10424287; doi:10.1111/jcmm.17813)

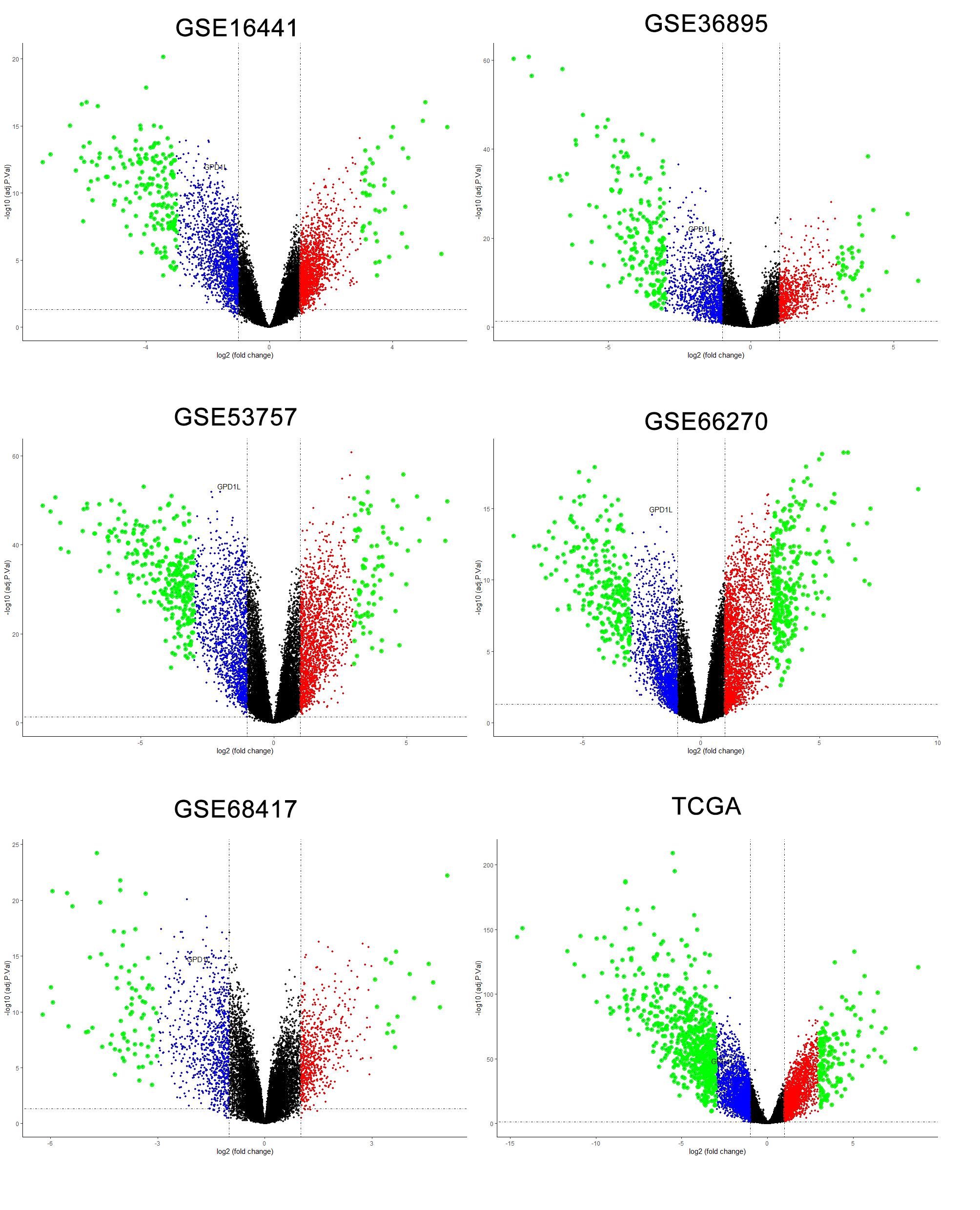

Supplement: Supplementary file 1 — Figure S1 [file JCMM-27-2328-s006.tif]

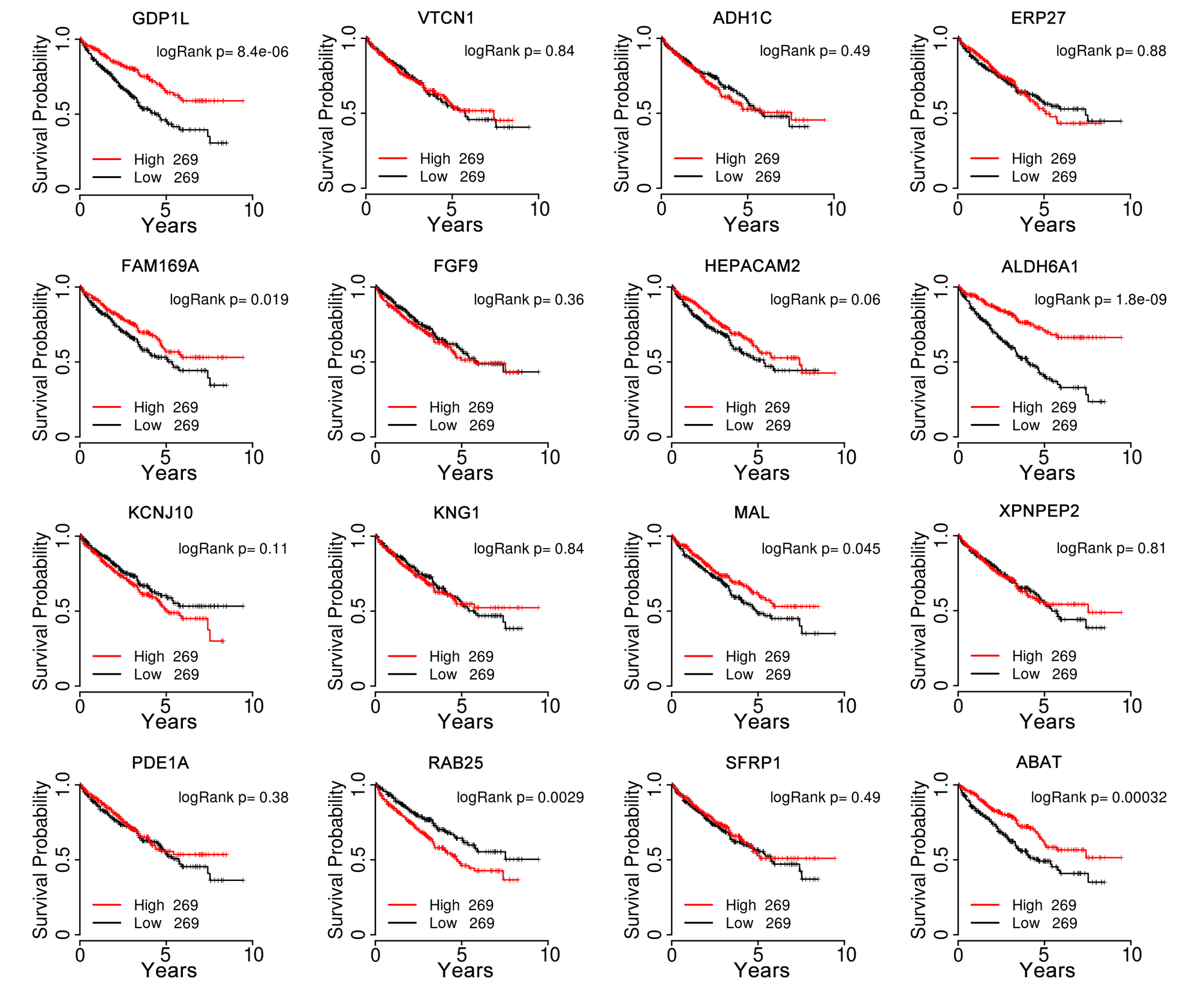

Supplement: Supplementary file 2 — Figure S2 [file JCMM-27-2328-s005.tif]

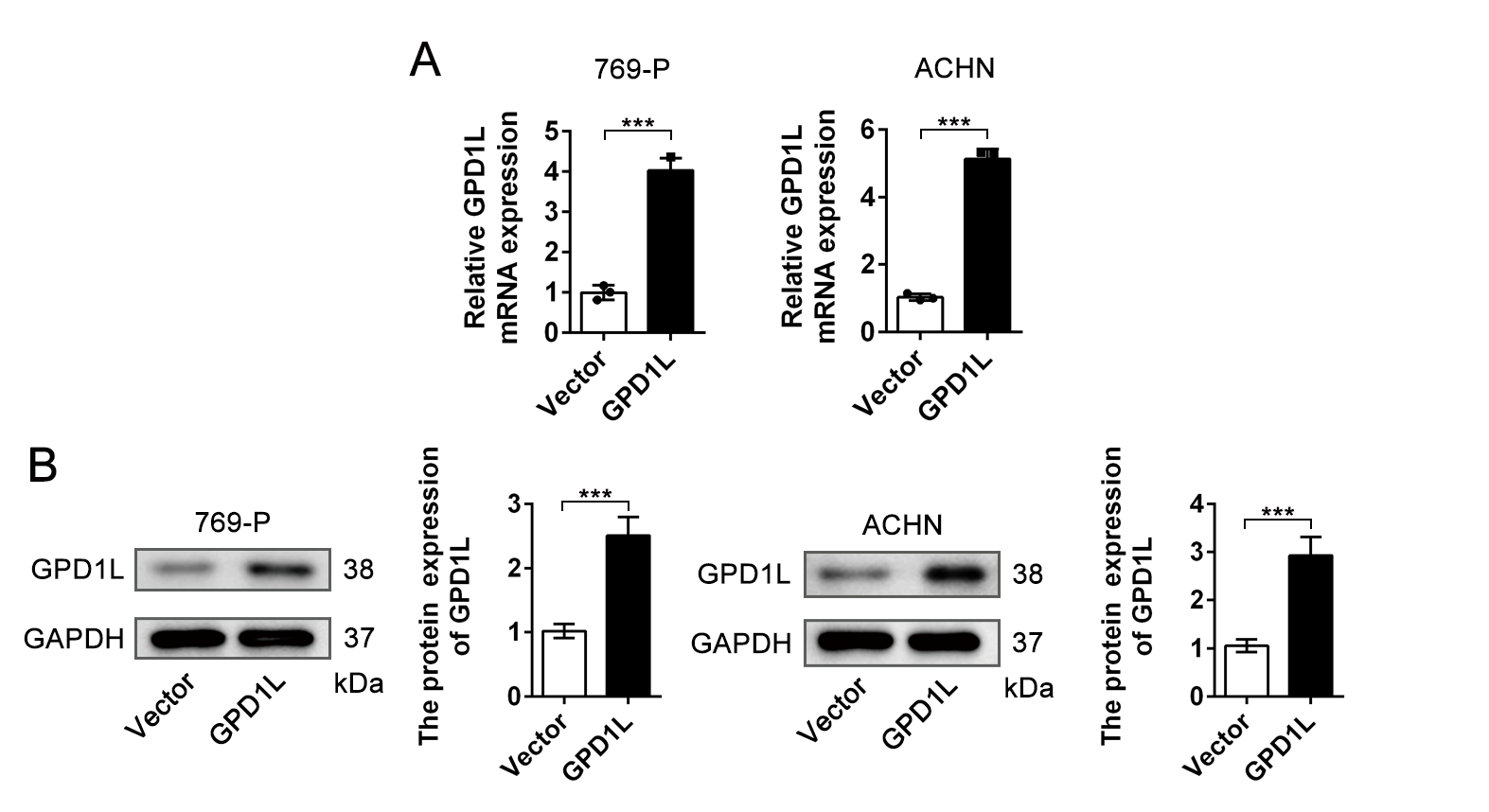

Supplement: Supplementary file 3 — Figure S3 [file JCMM-27-2328-s002.tif]

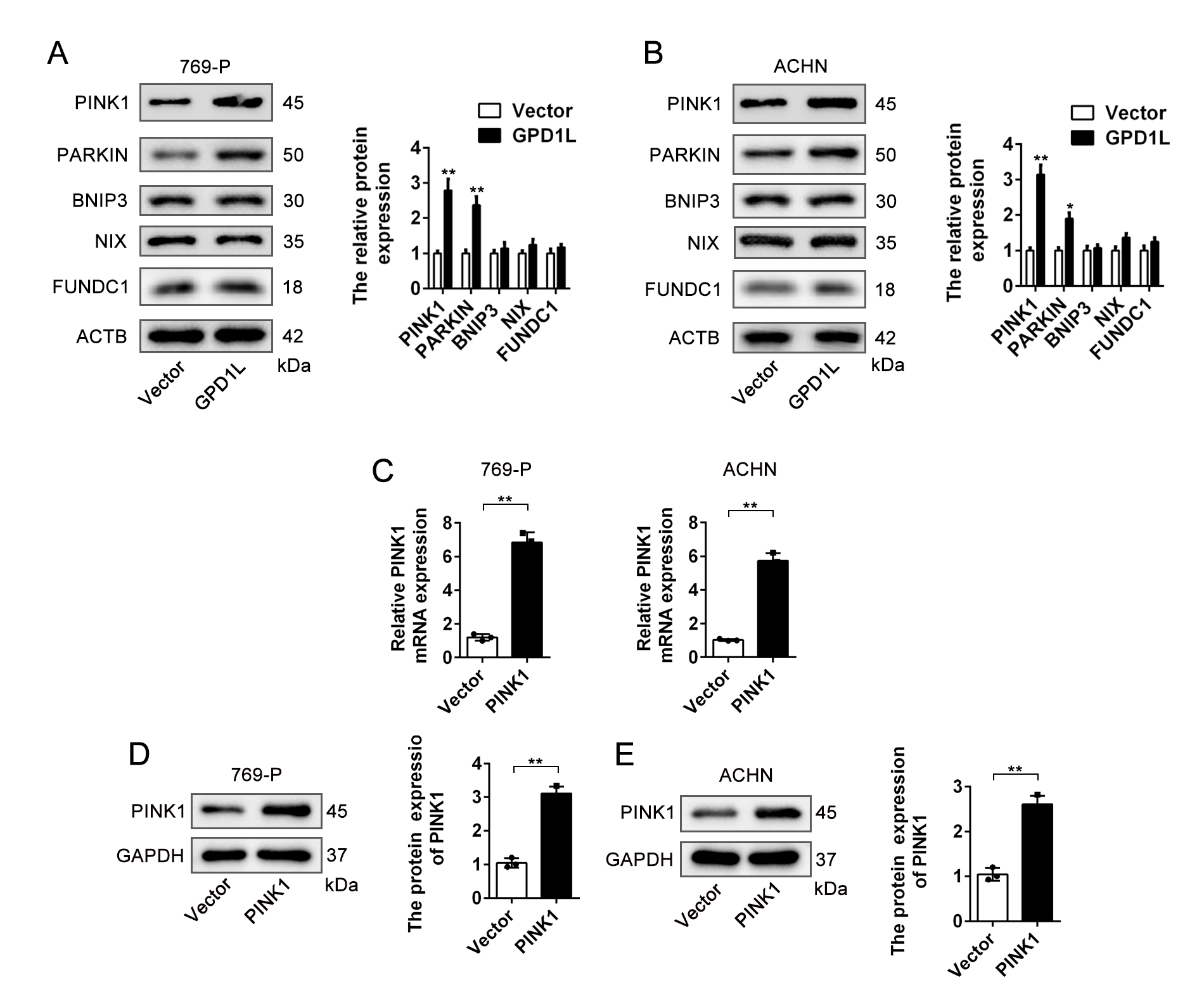

Supplement: Supplementary file 4 — Figure S4 [file JCMM-27-2328-s001.tif]

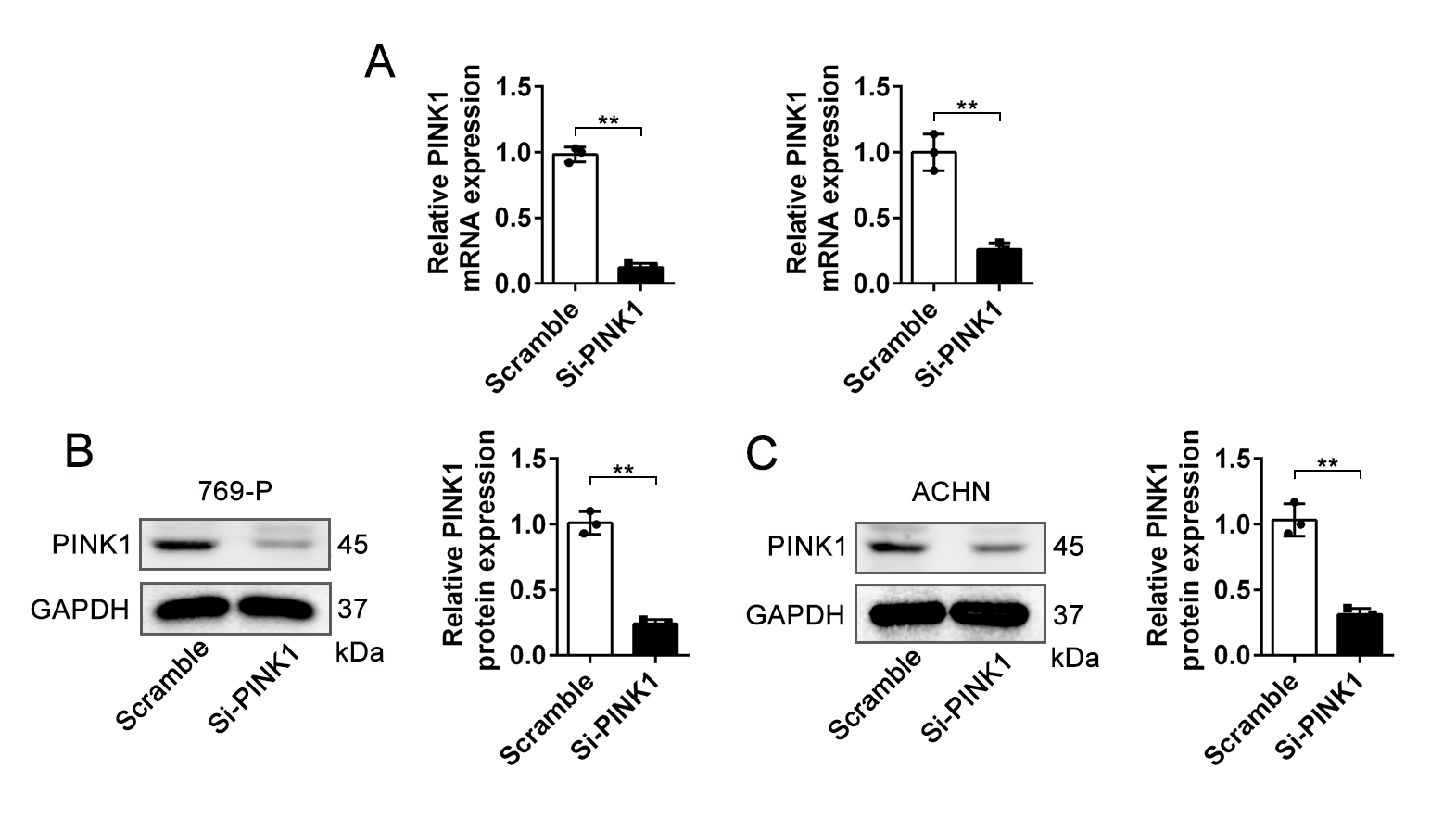

Supplement: Supplementary file 5 — Figure S5 [file JCMM-27-2328-s003.tif]
