# Supplementary material for: GPD1L inhibits renal cell carcinoma progression by regulating PINK1/Parkin‐mediated mitophagy
Source: J Cell Mol Med. 2023 Jun 29;27(16):2328–39. doi: 10.1111/jcmm.17813 (PMC10424287; doi:10.1111/jcmm.17813)
Supplement: Supplementary file 6 — Table S1 [file JCMM-27-2328-s004.docx]

Table S1. Antibody used in this study.

| Antibody | WB | IF | Company |
| --- | --- | --- | --- |
| GPD1L | 1:1000 | 1:50 | SANTA CRUZ |
| Bax | 1:1000 |  | Cell Signaling Technology |
| Bcl2 | 1:1000 |  | Abcam |
| TOM20 |  | 1:500 | Abcam |
| TIM23 | 1:2000 |  | Abcam |
| PINK1 | 1:1000 | 1:100 | Proteintech |
| Parkin | 1:1000 |  | Cell Signaling Technology |
| MFN2 | 1:1000 |  | Cell Signaling Technology |
| LC3B | 1:1000 | 1:200 | Abcam |
| P62 | 1:1000 |  | Cell Signaling Technology |
| GAPDH | 1:5000 |  | Abcam |
