# Supplementary material for: GPD1L inhibits renal cell carcinoma progression by regulating PINK1/Parkin‐mediated mitophagy
Source: J Cell Mol Med. 2023 Jun 29;27(16):2328–39. doi: 10.1111/jcmm.17813 (PMC10424287; doi:10.1111/jcmm.17813)
Supplement: Supplementary file 7 — Table S2 [file JCMM-27-2328-s007.docx]

Table S2 Primers designed for real-time PCR (Human)

| **Gene name** | **Forward** | **Reverse** | |
| --- | --- | --- | --- |
| GPD1L | GTTGCCATGTCAAATCTTAGCG | | GCACTCTCCCAGTGATCTCAT |
| PINK1 | GCCTCATCGAGGAAAAACAGG | | GTCTCGTGTCCAACGGGTC |
| Parkin | GTGTTTGTCAGGTTCAACTCCA | | GAAAATCACACGCAACTGGTC |
| BNIP3 | CAGGGCTCCTGGGTAGAACT | | CTACTCCGTCCAGACTCATGC |
| NIX | TTGGATGCACAACATGAATCAGG | | TCTTCTGACTGAGAGCTATGGTC |
| FUNDC1 | CCTCCCCAAGACTATGAAAGTGA | | AAACACTCGATTCCACCACTG |
| ACTB | CATGTACGTTGCTATCCAGGC | | CTCCTTAATGTCACGCACGAT |
